# Controllable synthesis of sphere-shaped interconnected interlinked binder-free nickel sulfide@nickel foam for high-performance supercapacitor applications

**DOI:** 10.1038/s41598-022-18728-1

**Published:** 2022-08-24

**Authors:** Batool Taher Al-Abawi, Nazish Parveen, Sajid Ali Ansari

**Affiliations:** 1 Department of Chemistry, College of Science, King Faisal University, P.O. Box 380, Hofuf, 31982 Al-Ahsa Saudi Arabia; 2 Department of Physics, College of Science, King Faisal University, P.O. Box 400, Hofuf, 31982 Al-Ahsa Saudi Arabia

**Keywords:** Chemistry, Engineering, Materials science, Nanoscience and technology

## Abstract

The fabrication of energy storage electrode materials with high specific capacitance and rapid charge–discharge capability has become an essential and major issue of concern in recent years. In the present work, sphere-shaped interconnected interlinked binder-free nickel sulfide (NiS) grown on the surface of a three-dimensional nickel foam (3DNF) was fabricated by a one-step solvothermal method under optimized synthesis conditions, including different solvents, amounts of sulfur, and experimental reaction times. The fabricated binder-free SS-NiS@3DNF-E electrodes were characterized by a range of spectroscopic and microscopic techniques and further evaluated for their comparative electrochemical supercapacitive performance in half-cell assembly cells. The optimized sphere-shaped interconnected interlinked binder-free SS-NiS@3DNF-E-3 electrode showed an outstanding specific capacitance of 694.0 F/g compared to SS-NiS@3DNF-E-1 (188.0 F/g), SS-NiS@3DNF-E-2 (470.0 F/g), and SS-NiS@3DNF-E-4 (230.0 F/g) as well as excellent cycling stability up to 88% after 6700 continuous charge–discharge cycles, with an energy density of 24.9 Wh/kg at a power density of 250.93 W/kg. The obtained results demonstrate that the interconnected interlinked binder-free NiS@nickel electrode is a potential candidate for energy storage applications.

## Introduction

In recent years, due to the depletion of fossil fuels, increase in the energy demand for vehicle power applications, and growing market of small electronic devices, environmental issues such as pollution and climate change have become more prominent^1,2^. Therefore, society is turning to sustainable and renewable energy sources, including solar energy, wind power, and geothermal energy^1–4^. These sources, however, are limited to specific times, conditions, such as sunny or rainy, and locations. As a result, clean energy conversion and storage technologies, including batteries, electrochemical supercapacitors (ESs), and fuel cells, have received much attention. These technologies are used in tablets, smartphones, cameras, and hybrid vehicles, and they play an important role as energy sources in daily life^1–6^.

Supercapacitors (SCs) have recently attracted much attention in the power field due to their high power density, outstanding cycling stability, fast charge/discharge process, and low cost. In addition, the arrangement of an SC between a battery with high energy storage capacity and a traditional capacitor with high energy density results in better electrochemical performance in a variety of applications^5,6^. ESs are employed in devices that can store a significant quantity of energy in a short period, such as hybrid platforms for trucks and buses, wind turbine and solar renewable energy systems, pulsed laser technology, and mobile phones^1,2,6^. The first ESs were reported in a patent filed by Beaker in 1957, which specified a capacitor based on large surface area carbon^7^. SCs are classified into three types based on the energy storage mechanism: electric double-layer capacitors (EDLCs), pseudocapacitors, and hybrid SCs, which combine both types of capacitors. In EDLCs, energy is stored through an adsorption/desorption process in which the ions of the electrode electrostatically interact with the electrolyte^6,8^. In pseudocapacitors, the charge is stored through a redox reaction that occurs during the charge/discharge process^9–12^. In addition, SCs are categorized as symmetric or asymmetric based on the electrode configuration when identical or different electrode materials are used^8,9,13^. The performance of SCs is strongly influenced by the materials used in the electrodes. Carbon-based materials, such as activated carbon (AC), graphene, and carbon nanotubes (CNTs), are utilized in EDLCs because they have a long cycle life and strong electrical conductivity, although their capacitance is usually low^1,5^. In contrast, pseudocapacitors mostly use metal oxides (MOs) and conducting polymers^1,5^. Ruthenium oxide (RuO_2_) is the most investigated electrode material due to its high specific capacitance, long cycle life, and high ionic conductivity. Its usage in SCs is restricted, however, due to its high cost and toxicity^14^. Therefore, transition MOs and their hydroxides are used as alternative materials, such as manganese oxide (MnO_2_), nickel oxide (NiO), Ni(OH)_2_, cobalt oxide (Co_3_O_4_), Co(OH)_2_, and vanadium oxide (V_2_O_5_)^1–4,14^. Because of their low cost, eco-friendliness, good theoretical specific capacitance, and low resistance, these materials are useful for constructing high energy density devices. However, transition metal hydroxides/oxides suffer from poor cycling stability and low conductivity, which result in decreased electron transport and relatively low theoretical capacity^2,3^. Recently, there has been a significant increase in research into transition metal sulfides (TMSs), such as CoS_2_, FeS_2_, MnS, CuS, and NiS, as promising materials for SC electrodes^8,11,15,16^ because of their advantages over their oxides, such as cost effectiveness, low environmental impact, excellent electrical conductivity, various valence states that provide sites for electrochemical activity, and higher capacitance^16^. Moreover, the shape, size, and morphology of metal sulfide-based electrode materials can influence their electrochemical performance. The electrodes based on metal sulfides and hydroxides operate on the faradic reaction mechanism as well as store energy over the electrode surface through reversible redox reactions, which is the main reason why they provide better electrochemical performance than the double-layer-based capacitors^16^. For example, a MnS composite with reduced graphene oxide (rGO) has been evaluated for use in SCs, and this electrode produces long cycling stability and high specific capacitance^17^. α-MnS/N-rGO was designed by Quan et al.^18^ as a cathode electrode material in asymmetric SC applications and delivered an energy density of 27.7 Wh kg^-1^ at a power density of 800 W kg. Copper sulfide (CuS) has been used as an electrode material, but its applications are limited by its low energy density and poor cycling stability, which must be improved^19^. To solve these problems, introducing a carbon material, which has a high power density and good cycling stability, into CuS preparation can enhance the performance of SCs^20^. BoopathiRaja et al.^21^ stated that a CuS/rGO composite electrode results in excellent long-term cycling stability (97% retention) and exhibits a 1604 Fg^-1^ capacitance at a current density of 2 Ag^-1^. Among all the above metal-based sulfides, NiS in particular has attracted considerable attention in the fields of energy storage, including batteries and SCs, due to its excellent physical and chemical properties and different phases, such as Ni_7_S_6_, Ni_9_S_8_, α-NiS, β-NiS, Ni_3_S_4_, and NiS_2_, most of which exist at room temperature^22–24^. Among these phases, NiS (α-NiS, β-NiS) is the most stable and sulfur-rich combination among the forms of nickel sulfides^22,23^. The phases are affected by temperature, and α-NiS with a rhombohedral crystal structure appears at low temperatures^22^, while β-NiS in a hexagonal form occurs at high temperatures^23,24^. Therefore, NiS is chosen for electrode purposes based on its phase diagram, which shows a rich sulfur structure and phase stability at normal temperature, which are the major points to consider when using NiS as a next-generation energy storage-based electrode material. For these reasons, NiS has been used as an effective electrode material due to its strong characteristics, including high conductivity, thermal stability, and slower volumetric expansion during the charge–discharge process^22,24^. Few works have been reported on NiS with different morphologies, such as that of Bhagwan et al*.*^25^, who synthesized β-NiS (3D) microflower electrodes with hierarchical geometries using a hydrothermal method, which exhibited high cycling stability and a specific capacitance of 1529 F/g. Similarly, Naresh et al.^22^ prepared NiS on nickel foam by hydrothermal treatment and investigated NiS with different morphologies by varying the reaction time and studying its role in the capacitive performance. Parveen et al.^6^ reported that hydrothermally synthesized shape-controlled hierarchical flower-like nickel sulfide exhibited an excellent specific capacitance of 603.9 F/g with a high cycling retention of 89% in aqueous electrolytes. Guan et al.^16^ and Balakrishnan et al.^26^ synthesized microflower-like NiS via a solvothermal method using Ni(OH)_2_ as precursors. The fabricated electrode delivered a 1122.7 Fg^-1^ specific capacitance at a 1 Ag^-1^ current density and excellent cycling stability after 100 cycles. Due to the unstable structure and effective sulfur content in the NiS system, the electrochemical properties (capacitance, cycling stability) of NiS in SCs are restricted. Subsequently, to address these issues, researchers attempted to synthesize NiS with varying sulfur concentrations under different conditions and study the impacts on the morphology to achieve excellent performance.

In the present work, a detailed study of the morphological changes of binder-free NiS@nickel foam and its effects on the ES performance is presented. NiS is grown on a three-dimensional conducting substrate via a simple and cost-effective solvothermal method and can act as a binder-free high-performance electrode material for SC applications. Different morphologies can be obtained through different solvents and by varying the amount of sulfur during the synthesis procedure. The fabricated sample with sphere-shaped interconnected interlinked binder-free NiS grown at the surface of 3DNF using 0.15 mM thiourea as the sulfur precursor is abbreviated as the SS-NiS@3DNF-E-1 electrode. The fabricated sample with sphere-shaped interconnected interlinked binder-free NiS grown at the surface of 3DNF using 0.80 mM thiourea as the sulfur precursor is abbreviated as the SS-NiS@3DNF-E-2 electrode. The fabricated sample with sphere-shaped interconnected interlinked binder-free NiS grown at the surface of 3DNF using 1.50 mM thiourea as the sulfur precursor is abbreviated as the SS-NiS@3DNF-E-3 electrode. The fabricated sample with sphere-shaped interconnected interlinked binder-free NiS grown at the surface of 3DNF using 2.50 mM thiourea as the sulfur precursor is abbreviated as the SS-NiS@3DNF-E-4 electrode. The electrochemical properties of the synthesized electrodes were evaluated using a three-electrode system by cyclic voltammetry (CV) and the galvanostatic charge–discharge (GCD) method in a 2 M KOH electrolyte solution. The optimized SS-NiS@nickel electrode showed an outstanding specific capacitance of approximately 694.0 F/g with an excellent cycling stability (88%) after 6700 continuous charge–discharge cycles.

## Results and discussion

### Effect of the solvent on morphological changes in SS-NiS@3DNF electrodes

**Figure 1:**
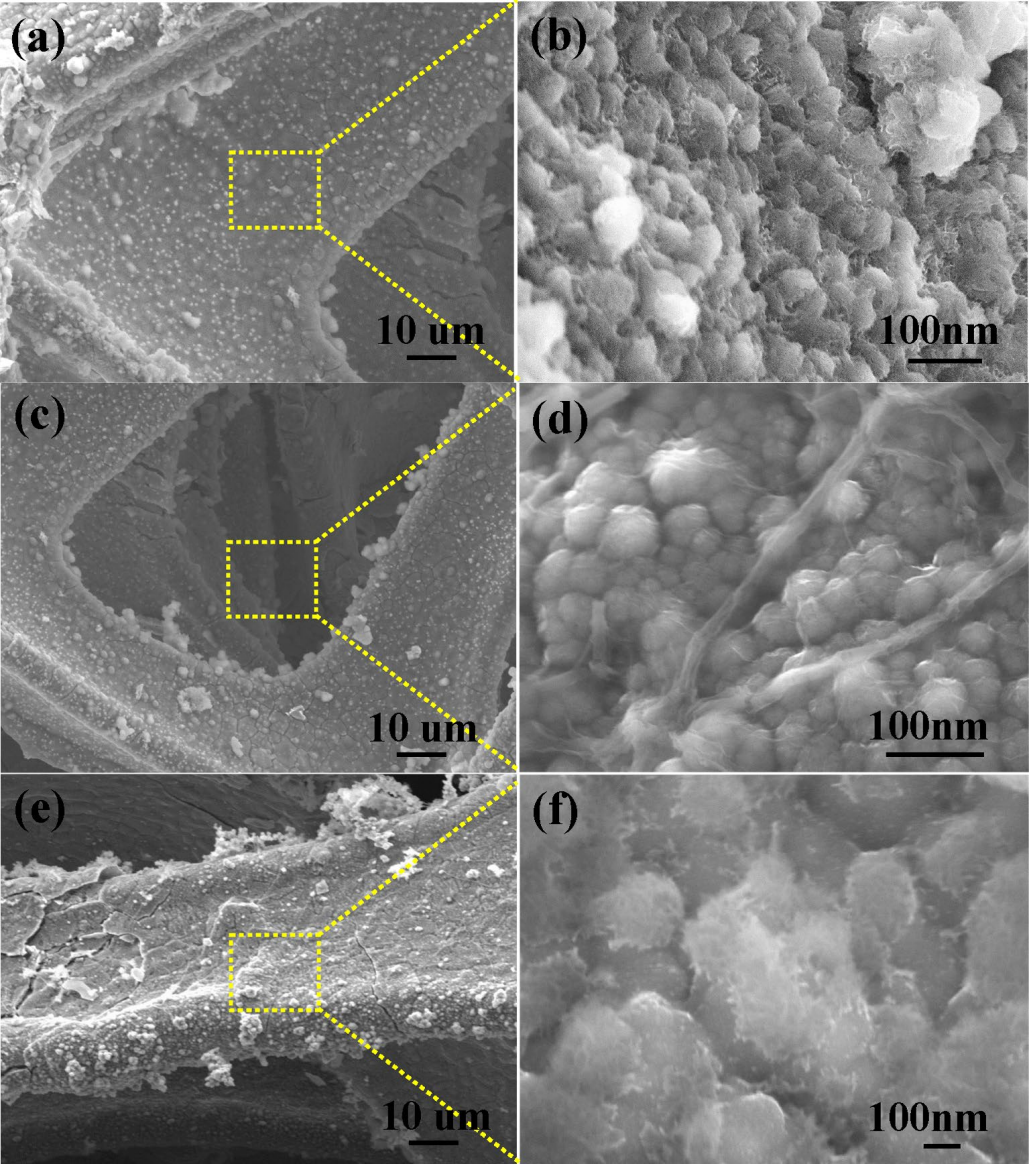
SEM images of the (**a**–**c**) SS-NiS@3DNF-M electrode, (**d**–**f**) SS-NiS@3DNF-E electrode, and (**g**–**i**) SS-NiS@3DNF-P electrode.

To understand the solvent effect on the morphology of SS-NiS@3DNF electrode nanostructures, we investigated this effect by changing the solvent during synthesis at fixed ethylene glycol amount, reaction temperature, and reaction time. When the synthesis proceeds in the presence of methanol, a few-layered sheet-like morphology of the NiS grown on a conductive 3DNF (Supplementary Fig. S1 online) substrate (SS-NiS@3DNF-M, Fig. 1a) is observed. At high magnification, the SEM image displayed in Fig. 1b shows that the SS-NiS@3DNF-M (area marked by the yellow dotted line) is composed of few-layered nanosheets with a diameter of 2–3 µm and has macro- and microsized pores inside the structure. In contrast, when the solvent is changed from methanol to ethanol during the solvothermal experiment, the SEM image of the SS-NiS@3DNF-E electrode shows an irregular spherical morphology (Fig. 1c) with a size of 10 µm to 15 µm (Fig. 1d). However, at higher magnification, the SEM image (Fig. 1d) of SS-NiS@3DNF-E shows that each sphere is interconnected and interlinked to each other, which is beneficial for contact of the electrolyte and active electrode material surface during the electrochemical process. In addition, the SS-NiS@3DNF-P electrode displays an aggregated sphere-shaped morphology (Fig. 1e and f).

**Figure 2:**
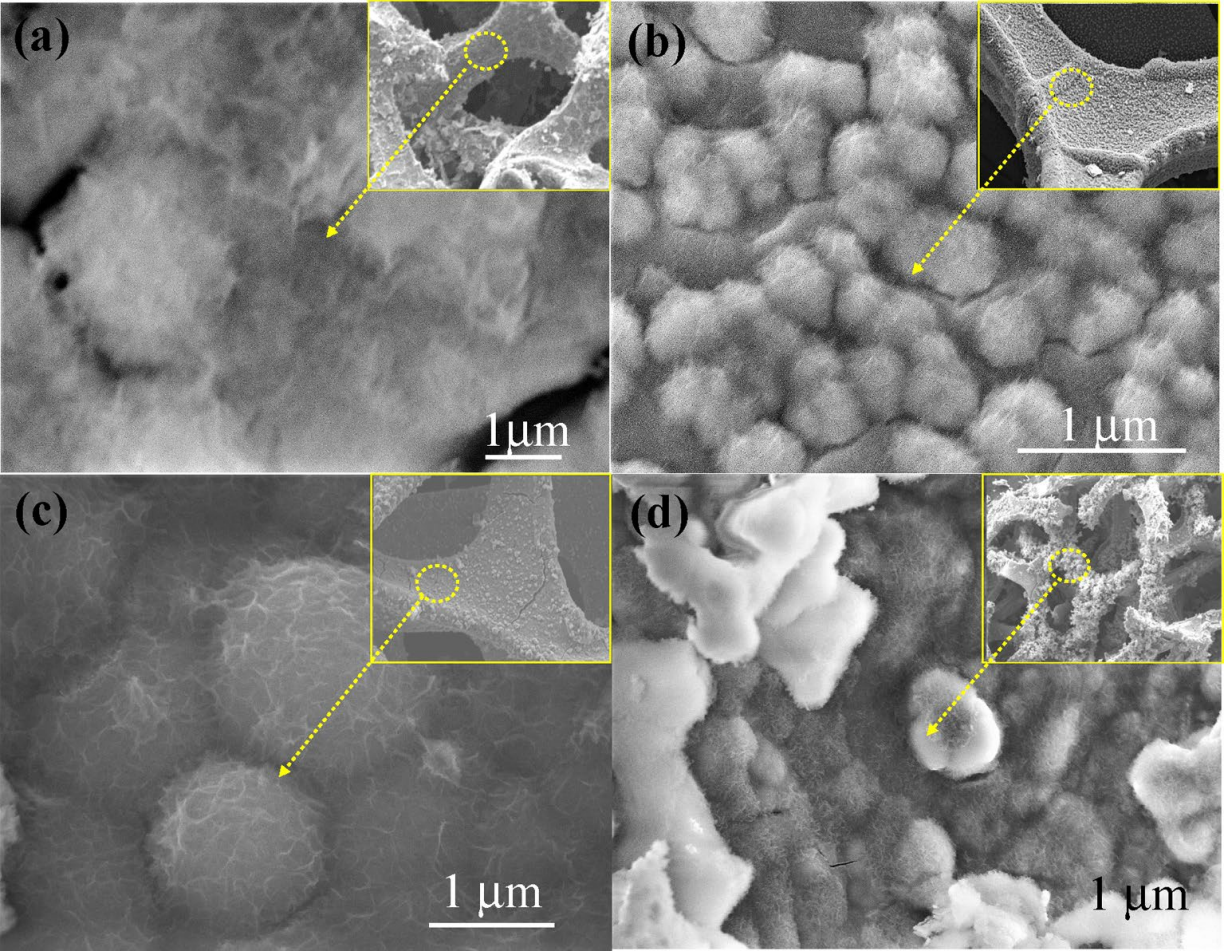
High- and low-magnification SEM images of (**a**) SS-NiS@3DNF-E-1, (**b**) SS-NiS@3DNF-E-2, (**c**) SS-NiS@3DNF-E-3, and (**d**) SS-NiS@3DNF-E-4.

SEM images of the other electrode, i.e., SS-NiS@3DNF-E-1, SS-NiS@3DNF-E-2, SS-NiS@3DNF-E-3, and SS-NiS@3DNF-E-4 prepared using different amounts of thiourea as the sulfur precursor, were also examined by FESEM, and the results are displayed in Fig. 2. The SEM analysis results show that thiourea played an important role in controlling the morphology and size of the nickel sulfide grown at the surface of 3DNF; thiourea is a very inexpensive sulfur precursor and is easily available compared to other sulfur-based compounds^27^.

Figure 2a shows an SEM image of the SS-NiS@3DNF-E-1 electrode, which shows an aggregate interconnected sheet-like structure deposited on 3DNF, whereas an irregular sphere-shaped morphology was observed in the case of the SS-NiS@3DNF-E-2 electrode (Fig. 2b). With a further increment of the thiourea concentration from 0.15 to 1.5 mM, the morphology of the SS-NiS@3DNF-E-3 electrode remains the same as that of the SS-NiS@3DNF-E-2 electrode, but the size of the spherical particles entirely changes into a spherical morphology with a uniform structure between 2 and 4 µm in size (Fig. 2c and Supplementary Fig. S2 online). The figure shows that all sphere shapes were interconnected and interlinked to each other, which helped with contact of the electrolyte and active electrode material surface during the electrochemical supercapacitive process. Figure 2d shows an SEM image of the SS-NiS@3DNF-E-4 electrode, which displays irregular morphology and shape, and the shape is not very clear compared to the other electrodes. These results show that the appropriate concentration of thiourea played an important role in the formation of well-defined SS-NiS@3DNF, which is discussed in detail in the following synthesis mechanism section.

The above morphology was further examined by the TEM and HRTEM analysis which shows that the dark colored spheres interlinked with the other spheres through the thin sheets (Supplementary Fig. S3 online). The HRTEM images shows that the grown nickel sulfide at the surface of the three dimensional nickel foam has well-ordered crystalline structure which well matched with previously reported work ^22,25^.

### Proposed reaction mechanism of the SS-NiS@3DNF electrode

Morphological studies support the role of the solvent and the effect of the sulfur precursor concentration as follows. The nickel salt and thiourea were dissolved in an ethylene glycol and ethanol solvent, which subsequently formed strong complexation between nickel ions (Ni^2+^) and thiourea, leading to the formation of a nickel-thiourea complex compound, which subsequently decomposed during the thermal process. Additionally, thermal treatment prevents the production of a large number of S^2−^ ions in the solution, which provides favorable conditions for the formation of nickel sulfide products. The above statements can be expressed in terms of the following suggested reaction^27^:

$$Ni^{ + 2} + H_{2} NCSNH_{2} \frac{Ethylene\, glycol}{{Ethanol}} \to \left[ {Ni\left( {SCN_{2} H_{4} } \right)_{2} } \right]^{ + 2} Reaction 1$$

$$\left[ {Ni\left( {SCN_{2} H_{4} } \right)_{2} } \right]^{ + 2} \frac{Decomposition}{{in\, thermal\, condition}} \to NiS Reaction 2$$

**Figure 3:**
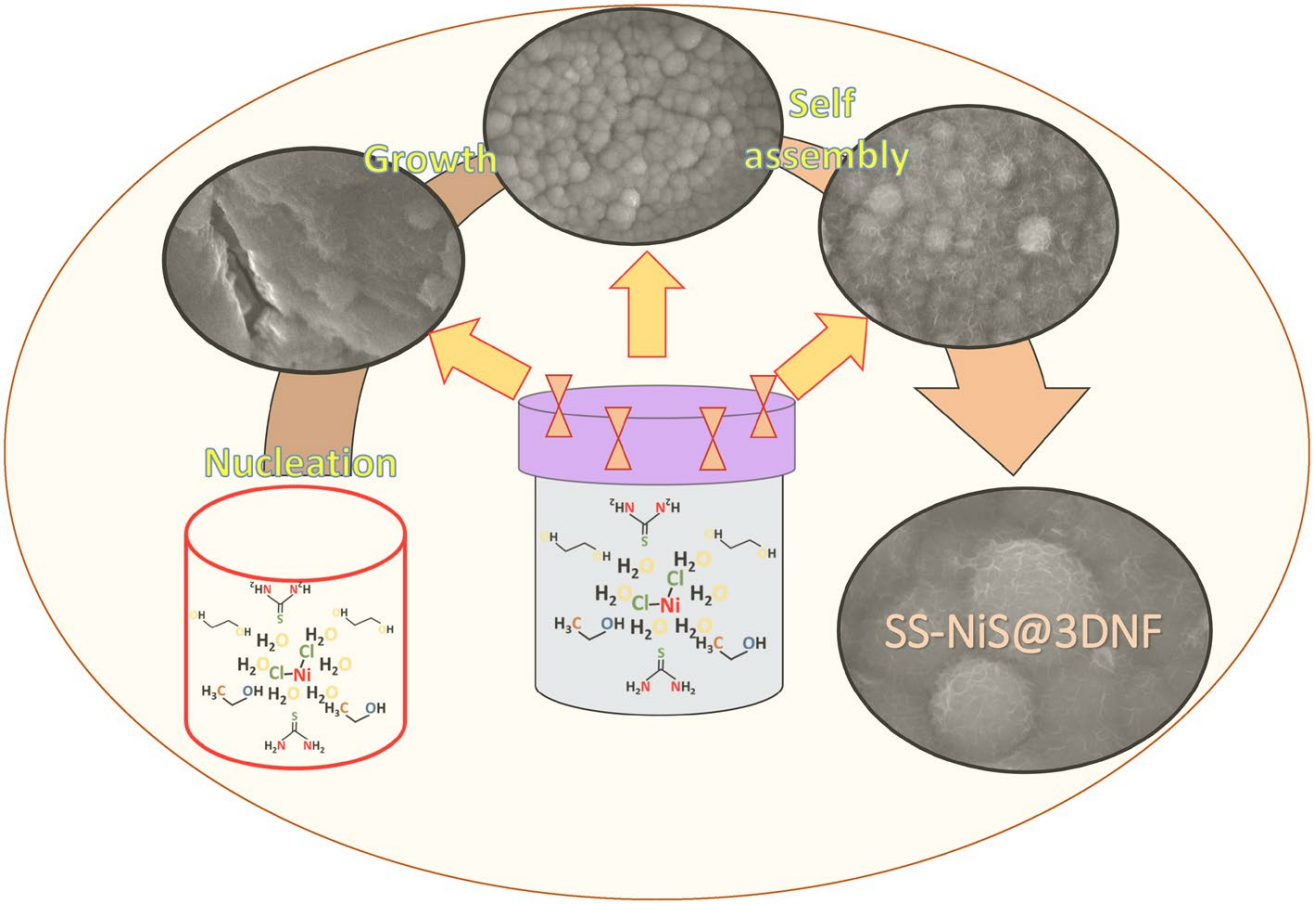
Schematic presentation of the SS-NiS@3DNF electrode fabrication.

The morphology of the SS-NiS@3DNF electrodes was significantly affected by various reaction parameters, such as the thiourea concentration, reaction time, and solvent effect, which are systematically shown in Fig. 3. The shape and size of the nanomaterial during synthesis have a significant effect on the reaction rate. In the present case, ethylene glycol and ethanol play an important role in the fabrication of SS-NiS@3DNF electrodes. Ethylene glycol and ethanol act as both reaction media and dispersion media and can efficiently absorb and stabilize the surface of the particles, producing monodisperse metal sulfide crystals with good dispersity^28^. However, ethylene glycol has a high permanent dipole moment and is an excellent susceptor of reactor heat during hydrothermal treatment; it can take energy, which helps decompose the thiourea and nickel complex compound ([Ni(SCN_2_H_4_)_2_]^2+^) and initiate the formation of the product on the provided substrate^29,30^. During the synthesis procedure, at the beginning of the reaction inside the Teflon reactor, frequent formation of nuclei started, and after time, the nuclei tended to aggregate (3 h Supplementary Fig. S4a online) with nonbearing nuclei, leading to the formation of a spherical shell (6 h, Supplementary Fig. S4b online) on 3DNF. After 12 h (Supplementary Fig. S4c online), the sphere-shaped nickel sulfide started to interconnect and interlink with the neighboring spheres.

After increasing the synthesis time (24 h, Supplementary Fig. S4d online), the spheres became larger in size, and the interconnection between spheres decreased compared to the SS-NiS@3DNF after 12 h. The growth procedure of the SS-NiS@3DNF electrode spheres was performed under controlled time and solution environment, resulting in interlinked-interconnected spheres of nickel sulfide grown on the nickel foam. When the concentration of thiourea was low, the reaction rate was low due to the low ability of sulfur ions to react with the crystal faces. Under low sulfur conditions, the nuclei tended to undergo isotropic growth and start forming a thermodynamically favorable spherical morphology. In contrast, when the concentration of thiourea was high, the nucleation rate was also high, and the consistent growth of nucleated particles was reduced; under this condition, the synthesized nickel sulfide was large in size.

**Figure 4:**
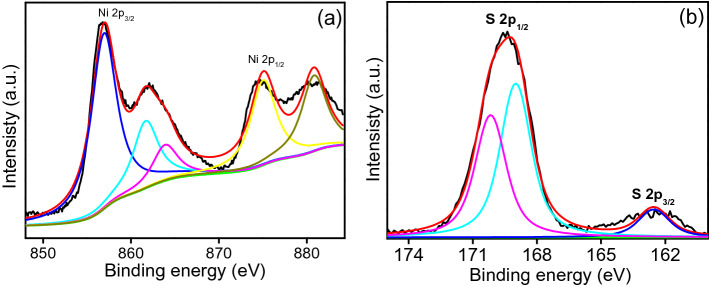
High-resolution XPS spectra of (**a**) Ni 2p and (**b**) S 2p of the SS-NiS@3DNF-E-3 electrode.

Supplementary Fig. S5 online shows the powder XRD pattern of the optimized SS-NiS@3DNF-E-3, which showed smooth lines with sharp peaks at 30.09°, 34.58°, 45.80,° 53.5°, 60.7°, 62.64°, 65.3°, 70.61° and 73.2° 2θ values corresponding to the (100), (101), (102), (110), (103), (200), (201), (004), and (202) planes, respectively. All the peaks of the optimized and fabricated SS-NiS@3DNF-E-3 electrode were indexed and well matched JCPDF No: 10–075-0613^6,22^. XPS was also performed to determine the chemical composition and surface characteristics, such as the surface percentage and nature of nickel and sulfur bonds, of the optimized SS-NiS@3DNF-E-3 material (Supplementary Fig. S6)^22,25^. Figure 4a shows the XPS high-resolution Ni 2p core level spectrum, which is divided into two broad peaks at binding energies of 871.80 and 853.3 eV assigned to Ni^2+^, while those observed at 855.85 and 874.20 eV correspond to Ni^3+^ and two shakeup satellite bands, which further demonstrate the presence of Ni 2p in SS-NiS@3DNF-E-3^25^. Figure 4b shows the high-resolution XPS S 2p spectrum, which contains two main peaks at binding energies of 160.0 to 164.0 eV, which shows that sulfur is present in the sulfide phase over the SS-NiS@3DNF-E-3 electrode^25^. The band observed at 168.2 eV was attributed to SO_3_. The presence of other sulfur species was still obviously expected because the surface of sulfide easily oxidized in ambient air and formed other forms of sulfur. However, the presence of high oxidation state sulfur does not appear to affect the electrochemical performance.

### Electrochemical measurement

**Figure 5:**
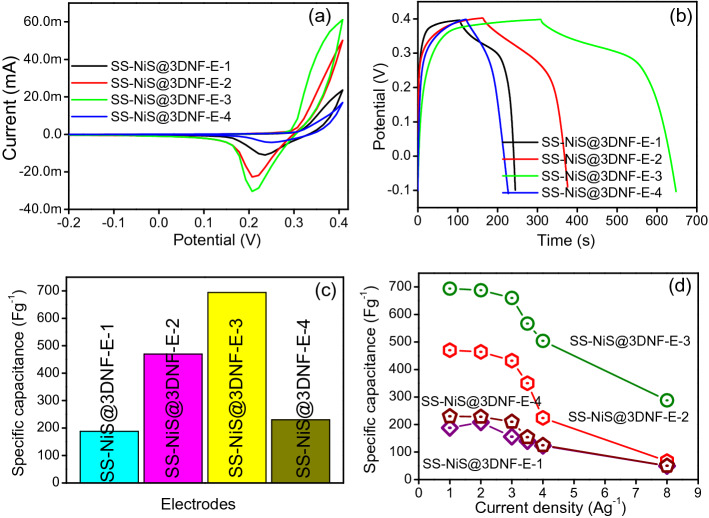
(**a**) Comparative CV graph of the SS-NiS@3DNF-E-1, SS-NiS@3DNF-E-2, SS-NiS@3DNF-E-3, and SS-NiS@3DNF-E-4 electrodes., (**b**) comparative GCD graph of the SS-NiS@3DNF-E-1, SS-NiS@3DNF-E-2, SS-NiS@3DNF-E-3, and SS-NiS@3DNF-E-4 electrodes., (**c**) comparative specific capacitance bar graph of the SS-NiS@3DNF-E-1, SS-NiS@3DNF-E-2, SS-NiS@3DNF-E-3, and SS-NiS@3DNF-E-4 electrodes., and (**d**) specific capacitance at different current densities of the SS-NiS@3DNF-E-1, SS-NiS@3DNF-E-2, SS-NiS@3DNF-E-3, and SS-NiS@3DNF-E-4 electrodes.

The comparative electrochemical performances of all the fabricated electrodes, i.e., SS-NiS@3DNF-E-1, SS-NiS@3DNF-E-2, SS-NiS@3DNF-E-3, and SS-NiS@3DNF-E-4, as possible binder-free electrodes for ES application were evaluated. The selection of the electrolyte is also an important parameter in electrochemical supercapacitive applications because its characteristics should include a high ionic concentration in a small amount of electrolyte and a low resistance. Therefore, KOH electrolyte is better than the other electrolytes owing to its low resistance and high ionic concentration. The initial electrochemical supercapacitive behavior of all the electrodes (SS-NiS@3DNF-E-1, SS-NiS@3DNF-E-2, SS-NiS@3DNF-E-3, and SS-NiS@3DNF-E-4) was analyzed by CV in a 2 M KOH electrolyte, and the results are displayed in Fig. 5a and Supplementary Fig. S7 online. Figure 5a shows the comparative CV graph of the SS-NiS@3DNF-E-1, SS-NiS@3DNF-E-2, SS-NiS@3DNF-E-3, and SS-NiS@3DNF-E-4 electrodes recorded at a fixed scan rate of 10 mV/s within the potential range of −0.2 to 0.4 V. Figure S 7 shows the CV graph of the SS-NiS@3DNF-E-1, SS-NiS@3DNF-E-2, SS-NiS@3DNF-E-3, and SS-NiS@3DNF-E-4 electrodes recorded at different scan rates within the potential range of -0.2–0.4 V. The comparative CV curves of all the electrodes (Fig. 5a) revealed that all the electrodes show the presence of the redox peak corresponding to the reversible faradic reaction over the electrode due to the possible reversible reaction from Ni-S to Ni-S-OH in the charge storage mechanism, which can be summarized as follows^22,25^:

$${\text{NiS }} + {\text{ OH}}^{ - } { \leftrightarrows }{\text{NiSOH }} + {\text{ e}}^{ - }$$

The observed peak at approximately 0.34 V was assigned to oxidation of Ni-S to Ni-S-OH, and the corresponding reduction peak at 0.2 V was attributed to the reversible reaction process. In addition, among all the electrodes, SS-NiS@3DNF-E-3 showed a high current response with a large integrated area compared to SS-NiS@3DNF-E-1, SS-NiS@3DNF-E-2, and SS-NiS@3DNF-E-4 in the CV results, which indicates that the electrochemical capacitive performance of the SS-NiS@3DNF-E-3 electrode may be higher than that of the rest of the electrodes. From the CV curves, we can clearly see that when we increased the thiourea amount, the CV integrated area became large for the SS-NiS@3DNF-E-1, SS-NiS@3DNF-E-2, and SS-NiS@3DNF-E-3 electrodes, whereas in the case of SS-NiS@3DNF-E-3, the CV integrated area decreased, which may be due to a decrease in the interconnectivity between neighboring spheres. The large CV integrated area might also be due to each sphere being interconnected and interlinked to each other, which helps the flow of electrons during the electrochemical process over the electrode during the electrochemical measurements. The anodic and cathodic peaks of all the electrodes were shifted to the right and left with increasing scan rate (Supplementary Fig. S7 online). The small shift indicates that a more reversible and faster charge transfer phenomenon occurs during electrochemical analysis. For better recognition of the charge storage mechanism and potential specific capacitance of the fabricated binder-free SS-NiS@3DNF electrodes, GCD analysis was performed in the potential range of −0.1 to 0.4 V, and the results are displayed in Fig. 5 and Supplementary Fig. S8 online. Figure 5b shows the comparative GCD curves of the SS-NiS@3DNF-E-1, SS-NiS@3DNF-E-2, SS-NiS@3DNF-E-3, and SS-NiS@3DNF-E-4 electrodes recorded at a fixed current density of 1 A/g, whereas Supplementary Fig. S8 online shows the GCD graph of the individual electrodes recorded at different current densities. The comparative GCD graph and individual electrode GCD curves revealed that all the electrodes exhibited a pseudocapacitive nature, which is also in accordance with the above CV results. The GCD graph also shows the presence of a voltage plateau from 0.31 to 0.40 V, again suggesting that the redox reaction plays an important role in the overall charge–discharge process occurring over the electrode surface during the electrochemical process.

The comparative GCD graph and specific capacitance graph (Fig. 5c and d) clearly reveal that the SS-NiS@3DNF-E-3 electrode shows a much better specific capacitance than the SS-NiS@3DNF-E-1, SS-NiS@3DNF-E-2, and SS-NiS@3DNF-E-4 electrodes, which may be due to all the spheres being interconnected and interlinked to each other, which provides a larger expose area and more electroactive sites for ions and electrons during the redox reaction. Additionally, direct growth of NiS on the conductive 3DNF substrate facilitates ionic and electronic transport, which enhances the overall performance of the SS-NiS@3DNF-E-3 electrode. Moreover, the specific capacitance of all the fabricated electrodes was calculated from the discharge curves using the equation mentioned in the electronic supplementary information, in which the highest specific capacitances of the SS-NiS@3DNF-E-1, SS-NiS@3DNF-E-2, SS-NiS@3DNF-E-3, and SS-NiS@3DNF-E-4 electrodes were 188.0, 470.0, 694.0 and 230.0 F/g at a current density of 1 A/g. Additionally, the GCD profiles of each electrode were also examined at different current densities. The specific capacitance of the SS-NiS@3DNF-E-1 electrode was 188.2, 180.4, 156.6, 140.0, 128.0, and 49.5 Fg^−1^ (Supplementary Fig. S8a online), whereas for the SS-NiS@3DNF-E-2 electrode, it was 470.0, 464.0, 432.0, 350.0, and 224.0 F/g (Supplementary Fig. S8b online). Similarly, for the SS-NiS@3DNF-E-3 electrode, it was 694.0, 780.0, 688.0, 660.0, 504.0, and 288.0 F/g (Supplementary Fig. S8c online), and for the SS-NiS@3DNF-E-4 electrode, the calculated specific capacitance was 230.0, 228.0, 210.0, 154.0, 112.0, and 48.0 F/g (Supplementary Fig. S8d online) at current densities of 1, 2, 3, 3.5, 4, and 6 A/g, respectively.

The GCD curves of all the individual electrodes and the corresponding specific capacitance performance at different current densities clearly show that with increasing current density, the restriction of the electron and electrolyte transport gradually decreases, which is responsible for the decrease in the capacitance of the electrode. The relationship between the current density and specific capacitance is presented in Fig. 5d. The specific capacitance of all the fabricated electrodes gradually decreased with increasing current density. This phenomenon occurs due to the internal voltage drop and insufficient active material involved in the redox reaction at higher currents. Furthermore, the specific capacitance of the optimized SS-NiS@3DNF-E-3 electrode is also compared with those of previously reported nickel sulfide-based electrode materials in detail in Table 1. The optimized SS-NiS@3DNF-E-3 electrode shows good specific capacitance even at high current density, which confirms the good rate capability of the optimized electrode.

**Table 1:** Comparison of the specific capacitance, energy density and power density of the SS-NiS@3DNF-E-3 electrode with those of other reported nickel sulfide-based electrode materials.

Electrode	Electrolyte	Specific capacitance	Retention	Energy and power density	Refs
NiS	2 M KOH	529 Fg^–1^ at 2 Ag^–1^	100% after 2000 cycles	–	^31^
NiS@SS	2 M KOH	441 Fg^–1^	–	10.1 Wh/kg at 4.5 kW/kg	^32^
3D Ni3S2	1 M KOH	626.1 Fg^–1^ at 5 Ag^–1^	2000 cycles	88.8 Wh/kg at 480 W/kg	^33^
β-NiS	2 M KOH	415.8 Fg^–1^ at 0.5 mAcm–2	91% after 2000 cycles	–	^34^
NiS/rGO	2 M KOH	530.1 Fg^–1^ at 4 Ag^–1^	90.90% after 2000 cycles	–	^35^
Ni3S2	2 M KOH	694 Fg^–1^ at 3.45 Ag^–1^	89.3% after 5000 cycles	1.96 mWhcm –3 at 0.6 W cm–3	^36^
Ni3S2/CNT	2 M KOH	514 Fg^–1^ at 4 Ag^–1^	88% after 1500 cycles	–	^37^
NiS-CFs	2 M KOH	635.1 Fg^–1^ at 1 Ag^–1^	96.4% after 5000 cycles	–	^38^
NiS/RGO	6 M KOH	305 Fg^–1^ at 1.1 Ag^–1^	91% after 3000 cycles	–	^39^
NiS@NF	6 M KOH	603.9 Fg^–1^ at 1 Ag^–1^	88.57% after 5000 cycles	–	^6^
rGO-Ni3S2	6 M KOH	616 Cg^–1^ at 1 Ag^–1^	92.7% after 5000 cycles	38.2 Wh/kg at 0.224 kW/kg	^40^
NiS2	3 M KOH	695 Fg^–1^ at 1.25 Ag^–1^	93.4% after 3000 cycles	15.7 Wh/kg at 254 W/kg	^41^
NiO/NiS	3 M KOH	386.7 Fg − 1 at 1 Ag^–1^	97.6% after 3000 cycles	–	^42^
SS-NiS@3DNF-E-3	2 M KOH	694 Fg^–1^ at 1 Ag^–1^	88% after 6700 cycles	24.9 Wh/kg at 250.93 W/kg	Present work

**Figure 6:**
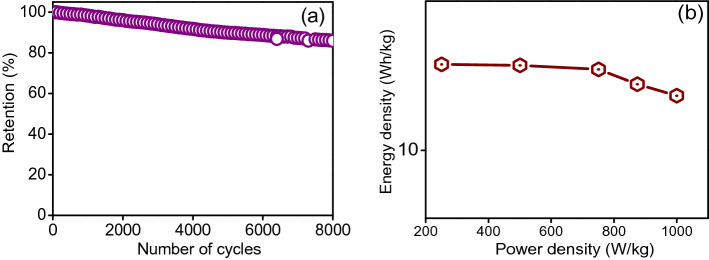
(**a**) Cyclic retention up to 6700 cycles, and (**b**) energy and power density curve of the SS-NiS@3DNF-E-3 electrode.

Long-term continuous charge–discharge cycling or the cycling stability of the electrode is a critical issue and an important parameter for practical SC applications because MO-based electrodes usually suffer from a lack of long-term stability due to electrode material degradation. The cycling stability of the optimized SS-NiS@3DNF-E-3 electrode was evaluated by continuous GCD measurements for up to 6700 cycles at a fixed current density of 3.0 A/g. According to the cycling stability curve (Fig. 6a), at the start of the cycling stability test, the specific capacitance of the electrode increased due to the self-activation effect, and after that, it started slightly decreasing and stabilized at more than 88% after 6700 cycles. The cycling stability results were also compared with previous results, and the cycling stability in the present case was significantly higher than that of the other reported nickel sulfide-based electrode materials in Table 1.

The energy density and power density are other major concerns for practical application of all electrodes in SC applications. The energy and power densities of the optimized electrode were calculated from the GCD curve using the equation mentioned in the electronic supplementary information at different current densities and plotted on the Ragone diagram shown in Fig. 6b. The Ragone plot shows that the highest energy density of the SS-NiS@3DNF-E-3 electrode was approximately 24.9 Wh/kg at a power density of 250.93 W/kg, and the electrode maintained an energy density of 7.5 Wh/kg at a power density of 1500 W/kg at the current load. The excellent electrochemical performance of the fabricated optimized binder-free SS-NiS@3DNF-E-3 electrode is due first to its interconnected interlinked structure between spheres, which helps provide a large exposed area and more electroactive sites during the redox reaction. Second, direct growth on Ni foam facilitates ionic and electronic transport, which enhances the performance of the electrode. Third, direct growth on the 3DNF substrate prevents binder and conductive additive adhesion, which decreases the resistance. These conditions create an effective and stable pathway for charge transfer during the electrochemical supercapacitive process.

## Conclusion

In the present work, a facile one-step solvothermal method was used to fabricate a binder-free sphere-shaped interconnected interlinked SS-NiS@3DNF-E-3 electrode with appropriate solvent selection, optimized sulfur precursor amount and satisfactory reaction time. The optimized SS-NiS@3DNF-E-3 showed excellent performance in KOH electrolyte. The highest specific capacitance of the optimized electrode was 694.0 F/g at 1 A/g with an excellent capacitance retention of 88% after 6700 cycles. The SS-NiS@3DNF-E-3 electrode delivered a maximum energy density of 24.9 Wh/kg at a power density of 250.93 W/kg. In the current study, the supercapacitive performance of the SS-NiS@3DNF-E-3 electrode opens a new way to design cost-effective capacitors with potential in various energy storage applications.

## Methods

### Materials

Nickel chloride hexahydrate (NiCl_2_.6H_2_O) and ethylene glycol (CH_2_OH)_2_ were purchased from Scharlau. Thiourea was obtained from BDH Chemicals Ltd., England. Other chemicals, such as ethanol methanol and propanol, were purchased from Sigma–Aldrich. Nickel foam (99.99% purity) was obtained from MTI Corporation, USA.

### Characterization

The morphological changes during the experiments were analyzed by field emission scanning electron microscopy (FESEM). The phase and purity of the fabricated electrode material were tested by X-ray diffraction (XRD, PAN analytical, X'pert-PRO MPD), and the chemical composition was examined by X-ray photoelectron microscopy (XPS, ESCALAB 250 XPS system, UK). The electrochemical properties, such as CV, charge–discharge (CD) and stability, were determined on a Metrohm Nova Auto lab electrochemical workstation.

### Electrochemical measurement

The electrochemical performance of all fabricated nickel sulfide electrodes was examined in a three-electrode system (half-cell system). Ag/AgCl, Pt, and a NiS@Ni foam sheet were used as reference, counter, and working electrodes, respectively. All of the electrochemical measurements were carried out on a Metrohm Nova Auto lab electrochemical workstation in 2 M KOH aqueous electrolyte solution. The specific capacitance of the binder-free NiS@Ni electrodes was calculated based on the mass loading during the solvothermal synthesis.

### Calculation

To estimate the specific capacitance of the fabricated NiS@Ni cathode electrodes inside the half-cell assembly, the following equation was used^6,16,26^:

1 $${\text{C}} = {\text{Idt}}/{\text{mdV}}$$

where C is the specific capacitance (Fg^−1^), I is the applied current, t is the discharge time, m represents the mass of active materials on the surface of the current collector, and dV is the applied potential window.

The power density and energy density were estimated from the following Eq.^6,16,22,26^:

2 $${\text{E}} = {1}/{\text{2 CV}}^{{2}}$$

3 $${\text{P}} = {\text{E}}/{\text{t}}$$

where C is the specific capacitance, V is the applied potential window and t is the discharge time of the device.

### Solvothermal synthesis of sphere-shaped interconnected interlinked binder-free NiS@3DNF electrodes

#### Synthesis of NiS@3DNF electrodes with different solvents

The fabrication of binder-free SS-NiS@3DNF using a simple solvothermal method was as follows. NiCl_2_.6H_2_O (0.63 mmol) and 1.97 mmol thiourea were added into an ethanol and ethylene glycol solvent (20 ml + 2 ml). The mixture was stirred for 15 min, after which two Ni foam sheets (1 cm*1 cm, Supplementary Fig. S1 online) were added and sonicated for 5 min. The above solution and Ni foam were transferred into a Teflon-linked autoclave and kept at 120 °C for 12 h. After the reaction was completed, the autoclave was naturally cooled at room temperature. The synthesized electrodes were washed with deionized water and ethanol several times to remove all ions and contaminants remaining on the fabricated electrode. The washed samples were dried in an oven at 80 °C for 24 h. The fabricated sample with sphere-shaped interconnected interlinked binder-free nickel sulfide (NiS) grown at the surface of three-dimensional nickel foam (3DNF) using ethanol as the solvent is abbreviated as the SS-NiS@3DNF-E electrode. To better understand the solvent effect on the morphology of the NiS grown at the surface of nickel foam, methanol and propanol were also used, and the other parameters were the same. The fabricated sample with sphere-shaped interconnected interlinked binder-free NiS grown at the surface of 3DNF using methanol as the solvent is abbreviated as the SS-NiS@3DNF-M electrode. Similarly, the fabricated sample with sphere-shaped interconnected interlinked binder-free NiS grown at the surface of 3DNF using propanol as the solvent is abbreviated as the SS-NiS@3DNF-P electrode. After performing SEM analysis, SS-NiS@3DNF-E showed a better morphology, so in this study, we chose ethanol as the solvent for further studies. Moreover, Supplementary Table S1 online shows a comparison of the precursor material, synthesis method and morphology of NiS@Ni in the present case with those in previously reported articles based on NiS synthesis.

#### Synthesis of SS-NiS@3DNF with different sulfur precursor amounts

The detailed synthesis procedure for direct synthesis of SS-NiS@3DNF electrodes using the solvothermal method was the same as that discussed above. However, in this experiment, different amount of thiourea (0.13, 0.65, 1.3, 1.97 mM) were used, and its effect on the morphology was observed, whereas the amount of the nickel precursor and the reaction temperature and time, i.e., 120 °C for 12 h, remained the same in all the synthesis processes. The fabricated sample with sphere-shaped interconnected interlinked binder-free NiS grown at the surface of 3DNF using 0.13 mM thiourea as the sulfur precursor is abbreviated as the SS-NiS@3DNF-E-1 electrode. The fabricated sample with sphere-shaped interconnected interlinked binder-free NiS grown at the surface of 3DNF using 0.65 mM thiourea as the sulfur precursor is abbreviated as the SS-NiS@3DNF-E-2 electrode. The fabricated sample with sphere-shaped interconnected interlinked binder-free NiS grown at the surface of 3DNF using 1.30 mM thiourea as the sulfur precursor is abbreviated as the SS-NiS@3DNF-E-3 electrode. The fabricated sample with sphere-shaped interconnected interlinked binder-free NiS grown at the surface of 3DNF using 1.97 mM thiourea as the sulfur precursor is abbreviated as the SS-NiS@3DNF-E-4 electrode.

## Supplementary Information

Supplementary Information.

## Data Availability

The dataset used and/and or analyzed during the current study available from the corresponding author on reasonable request.
