# Supplementary material for: Controllable synthesis of sphere-shaped interconnected interlinked binder-free nickel sulfide@nickel foam for high-performance supercapacitor applications
Source: Sci Rep. 2022 Aug 24;12:14413. doi: 10.1038/s41598-022-18728-1 (PMC9402625; doi:10.1038/s41598-022-18728-1)
Supplement: Supplementary file 1 — Supplementary Information. [file 41598_2022_18728_MOESM1_ESM.docx]

**Controllable synthesis of sphere-shaped interconnected interlinked binder-free nickel sulfide@nickel foam for high-performance supercapacitor applications**

Batool Taher Al Abawi,^1^* Nazish Parveen,^1^*, Sajid Ali Ansari^2^

^1^Department of Chemistry, College of Science, King Faisal University, P.O. Box 380, Hofuf, Al-Ahsa 31982, Saudi Arabia

^2^Department of Physics, College of Science, King Faisal University, P.O. Box 400, Hofuf, Al-Ahsa 31982, Saudi Arabia

***Corresponding author**

Emails: 217012748@student.kfu.edu.sa; nislam@kfu.edu.sa; Tel.: +966-13-589-6915

**Table S1:** Shows a comparison of the precursor material, synthesis method and morphology of NiS@Ni in the present case with those in previously reported articles based on NiS synthesis

| **Ref.** | **Method** | **Solvent** | **Precursors** | **Synthesis Electrode material** |
| --- | --- | --- | --- | --- |
| **1** | Hydrothermal | DI water | (Ni(NO_3_)_2_⋅6H_2_O), CH_4_N_2_S, NH_3_ | β-NiS |
| **2** | Hydrothermal | DI water | NiSO_4_·6H_2_O, C_2_H_5_NS, C_3_H_7_NO_2_S | NiS@NF |
| **3** | Chemical bath deposition | Ethanol | (Ni(NO_3_ )_2_ ·6H_2_O), CH_3_CSNH_2_) | NiS@Ti substrate |
| **4** | Chemical bath deposition | DI water | NiSO_4_, Na_2_S_2_O_3_·5H_2_O, HCl | NiS thin film |
| **5** | Sonochemical | DI water | Ni(CH_3_COO)_2_.2H2O, TEA, C_2_H_5_NS | NiS nanoparticles |
| **6** | sonochemical | DI water | Ni(CH_3_COO)_2_ · 4H_2_O, C_2_H_5_NS | NiS nanoparticles |
| **7** | laser irradiation | DI water | Ni (CH_3_COO)_2_·4H_2_O, CH_3_CSNH_2_, (HOCH_2_CH2)_3_N | NiS nanostructures |
| **8** | Mmicrowave-assisted | Ethylene glycol | Ni (CH_3_COO)_2_. 4H_2_O (NH_2_)_2_CS | NiS nanoparticles |
| **9** | Solvothermal | Ethanol | NiCl_2_.6H_2_O, CH_3_CSNH_2_, acetic acid | H-flower-like NiS |
| 10 | spray pyrolysis | DI water | Ni(NO_3_)_2_.6H_2_O, CS(NH_2_)_2_, acetic acid | NiS films on glass |

**FESEM image of the 3DNF**

**Figure S1**. FESEM image of the 3DNF.

SEM image of the SS-NiS@3DNF-E-3 electrode.

**Figure S2**. SEM image of the SS-NiS@3DNF-E-3 electrode.

**TEM and HRTEM images of the SS-NiS@3DNF-E-3 electrode**

**Figure S3**.TEM and HRTEM images of the SS-NiS@3DNF-E-3 electrode.

XRD pattern of the SS-NiS@3DNF-E-3 electrode.

**FESEM image of the SS-NiS@3DNF electrode developed at 3h, 6h, 12h, and 24h**

**Figure S4**. FESEM image of the SS-NiS@3DNF electrode developed at (**a**) 3h, (**b**) 6h, (**c**) 12h, and (**d**) 24h.

XRD pattern of the SS-NiS@3DNF-E-3 electrode.

**Figure S5**. XRD pattern of the FESEM image of the SS-NiS@3DNF-E-3 electrode.

XPS survey spectra of the SS-NiS@3DNF-E-3 electrode

**Figure S6**. XPS survey spectra of the SS-NiS@3DNF-E-3 electrode.

**CV graph of the SS-NiS@3DNF-E-1, SS-NiS@3DNF-E-2, SS-NiS@3DNF-E-3, and SS-NiS@3DNF-E-4 electrode at different scan rates**

**Figure S7**. CV graph of the (**a**) SS-NiS@3DNF-E-1, (**b**) SS-NiS@3DNF-E-2, (**c**) SS-NiS@3DNF-E-3, and (**d**) SS-NiS@3DNF-E-4 electrode at different scan rates.

G**CD graph of the SS-NiS@3DNF-E-1, SS-NiS@3DNF-E-2, SS-NiS@3DNF-E-3, and SS-NiS@3DNF-E-4 electrode at different current densities**

**Figure S8**. GCD graph of the (**a**) SS-NiS@3DNF-E-1, (**b**) SS-NiS@3DNF-E-2, (**c**) SS-NiS@3DNF-E-3, and (**d**) SS-NiS@3DNF-E-4 electrode at different current densities.

**References**

[1] R. Bhardwaj, R. Jha, M. Bhushan, and R. Sharma, “Comparative study of the electrochemical properties of mesoporous 1-D and 3-D nano- structured rhombohedral nickel sulfide in alkaline electrolytes,” *J. Phys. Chem. Solids*, vol. 144, no. February, p. 109503, 2020, doi: 10.1016/j.jpcs.2020.109503.

[2] B. Naresh, D. Punnoose, S. S. Rao, A. Subramanian, B. Raja Ramesh, and H. J. Kim, “Hydrothermal synthesis and pseudocapacitive properties of morphology-tuned nickel sulfide (NiS) nanostructures,” *New J. Chem.*, vol. 42, no. 4, pp. 2733–2742, 2018, doi: 10.1039/c7nj05054b.

[3] P. Gaikar, S. P. Pawar, R. S. Mane, M. Nuashad, and D. V. Shinde, “Synthesis of nickel sulfide as a promising electrode material for pseudocapacitor application,” *RSC Adv.*, vol. 6, no. 113, pp. 112589–112593, 2016, doi: 10.1039/c6ra22606j.

[4] A. M. Patil *et al.*, “Ultrathin nickel sulfide nano-flames as an electrode for high performance supercapacitor; Comparison of symmetric FSS-SCs and electrochemical SCs device,” *RSC Adv.*, vol. 6, no. 72, pp. 68388–68401, 2016, doi: 10.1039/c6ra12018k.

[5] H. Wang, J. R. Zhang, X. N. Zhao, S. Xu, and J. J. Zhu, “Preparation of copper monosulfide and nickel monosulfide nanoparticles by sonochemical method,” *Mater. Lett.*, vol. 55, no. 4, pp. 253–258, 2002, doi: 10.1016/S0167-577X(01)00656-5.

[6] M. Kristl, B. Dojer, S. Gyergyek, and J. Kristl, “Synthesis of nickel and cobalt sulfide nanoparticles using a low cost sonochemical method,” *Heliyon*, vol. 3, no. 3, pp. 1–19, 2017, doi: 10.1016/j.heliyon.2017.e00273.

[7] T. F. Hung, Z. W. Yin, S. B. Betzler, W. Zheng, J. Yang, and H. Zheng, “Nickel sulfide nanostructures prepared by laser irradiation for efficient electrocatalytic hydrogen evolution reaction and supercapacitors,” *Chem. Eng. J.*, vol. 367, pp. 115–122, 2019, doi: 10.1016/j.cej.2019.02.136.

[8] L. Peng *et al.*, “Nickel Sulfide Nanoparticles Synthesized by Microwave-assisted Method as Promising Supercapacitor Electrodes: An Experimental and Computational Study,” *Electrochim. Acta*, vol. 182, pp. 361–367, 2015, doi: 10.1016/j.electacta.2015.09.024.

[9] N. Parveen, S. A. Ansari, S. G. Ansari, H. Fouad, N. M. Abd El-Salam, and M. H. Cho, “Solid-state symmetrical supercapacitor based on hierarchical flower-like nickel sulfide with shape-controlled morphological evolution,” *Electrochim. Acta*, vol. 268, pp. 82–93, 2018, doi: 10.1016/j.electacta.2018.01.100.

[10] A. Gahtar, S. Benramache, A. Ammari, A. Boukhachem, and A. Ziouche, “Effect of molar concentration on the physical properties of NiS thin film prepared by spray pyrolysis method for supercapacitors,” *Inorg. Nano-Metal Chem.*, vol. 52, no. 1, pp. 112–121, 2022, doi: 10.1080/24701556.2020.1862225.
